# A Study of Histological and Clinical Parameters and Their Correlation With Lymph Node Metastasis and Two-Year Survival in 50 Cases of Oral Squamous Cell Carcinoma

**DOI:** 10.7759/cureus.59045

**Published:** 2024-04-26

**Authors:** Pretty Singh, Kavita Somani, Sujatha Poduwal, Garima Singh

**Affiliations:** 1 Department of Pathology and Laboratory Medicine, Apollomedics Superspeciality Hospital, Lucknow, IND

**Keywords:** oral squamous cell carcinoma, survival rate, lymph node metastasis, prognostic markers, histopathological spectrum

## Abstract

Introduction: Oral squamous cell carcinoma (OSCC) is one of the most prevalent malignant neoplasms in South Asia and a major public health problem in India. The purpose of the study was to identify correlations among various clinicopathological parameters of OSCC in a tertiary care center in the Eastern Uttar Pradesh population of North India. The study is imperative due to the scarcity of available data from this region.

Methodology: A retrospective observational study was conducted on the cases received in the Department of Pathology over the period of January 2021 to December 2021. The study analyzed cases of OSCC, focusing on various factors such as age, gender, habits, tumor site, tumor size, differentiation, tumor-stroma ratio, tumor-infiltrating lymphocytes, tumor budding, worst pattern of invasion, depth of invasion, perineural invasion, lymphovascular invasion, underlying bone and overlying skin involvement, regional lymph node metastasis, and overall two-year survival.

Results: The mean age of the patients was 47.80 ± 12.48 years, and the male-to-female ratio was 15.6:1. Buccal mucosa was the most frequently affected site followed by the tongue. Fifty-six percent of cases reported with a history of tobacco abuse. Thirty-six percent of the patients had regional lymph node metastasis and exhibited a strong association with younger age, substance abuse, higher tumor size, tongue as a site, moderate-to-poor tumor differentiation, low tumor-infiltrating lymphocytes, and higher perineural and lymphovascular invasion. Moreover, at the end of the two-year survival analysis, 34% of patients succumbed to the disease. Overall survival was observed to be significantly better with <2 cm maximum tumor size, well-differentiated tumor morphology, higher tumor-infiltrating lymphocytes, and no nodal metastasis.

Conclusions: The study highlights the intricate correlations of various histopathological factors in OSCC, shedding light on their potential implications for prognosis.

## Introduction

The prevalence of oral cancer is rapidly rising worldwide, making it a critical concern. It is responsible for a significant number of fatalities and has emerged as a prominent health issue in the present times [1]. More than 90% of malignancies of the oral cavity are squamous cell carcinoma arising from the mucosal epithelium [2]. According to the Global Cancer Observatory, there were an estimated 377,713 new cases of oral squamous cell carcinoma (OSCC) and 177,757 deaths worldwide in 2020 [3].

The consumption of tobacco in various forms is the primary cause of OSCC. Alcohol consumption and smoking are additional significant factors, while chronic irritation, poor oral hygiene, viral infection, occupational exposure, malnutrition, poor diet, and genetic factors are all potential risk factors [4].

The prevalence of OSCC in India carries great importance, as it contributes to a substantial proportion of worldwide cases, amounting to one-fourth of the total. Each year, approximately 77,000 new cases are detected, with 52,000 fatalities reported within the country [5]. The prevalence of oral cancer, especially in the northern region, can be attributed to the widespread consumption of gutka, a smokeless tobacco product, accompanied by the habit of chewing betel quid.

This high prevalence of OSCC in addition to the limited research conducted on this topic, especially in eastern Uttar Pradesh, necessitates further investigation into the prognostic factors.

## Materials and methods

This retrospective observational study was conducted in the Department of Pathology, in our tertiary care hospital. It included all biopsy-proven OSCC cases, who underwent radical resection with lymph node dissection in one year (January 2021-December 2021). A total of 50 cases were enrolled, following strict inclusion and exclusion criteria. In the inclusion criteria were all the biopsy-proven OSCC cases who underwent radical resection with lymph node dissection in our tertiary care center. The exclusion criteria included cases that had only undergone a small biopsy, patients who received prior treatment (chemotherapy/radiotherapy), and cases with recurrence.

Their comprehensive personal history and clinical and histological parameters were documented. Regional lymph node metastasis status was assessed, and patient outcome was noted as overall two-year survival. Patient details that were noted down were age, gender, habits/substance abuse, and the procedure that each patient had undergone. Furthermore, clinical parameters that were noted were as follows:

*Tumor site: *Tongue, buccal mucosa, retromolar area, upper alveolus and gingiva, lower alveolus and gingiva, floor of mouth, hard palate, upper or lower lip

*Tumor size:* Cases were segregated into three groups, based on the maximum tumor dimension into the following groups: ≤2 cm, >2 cm to ≤4 cm, and >4 cm. 

Histological details were noted on several well-prepared and hematoxylin-and-eosin-stained sections, ideally obtained from the innermost region of the tumor with progressive boundaries, sections of surgical margins, and sections of regional lymph nodes were examined under the microscope to analyze the subsequent histopathological parameters recognized as prognostic indicators.

The histological parameters studied are discussed next.

Tumor differentiation

**Figure 1:**
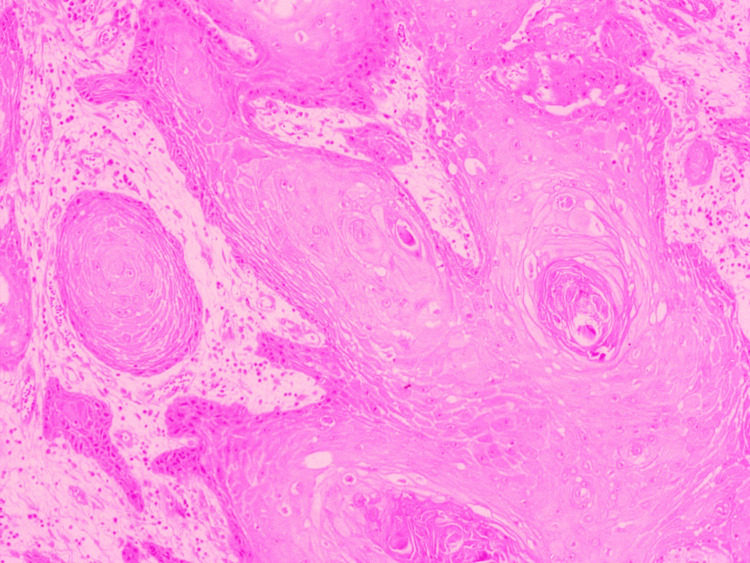
Hematoxylin and eosin: x400 squamous cell carcinoma - well differentiated.

The tumors were categorized as well-differentiated, moderately differentiated, and poorly differentiated according to the World Health Organization (WHO) Grading System (Figure 1) [6].

Tumor budding (TB)

**Figure 2:**
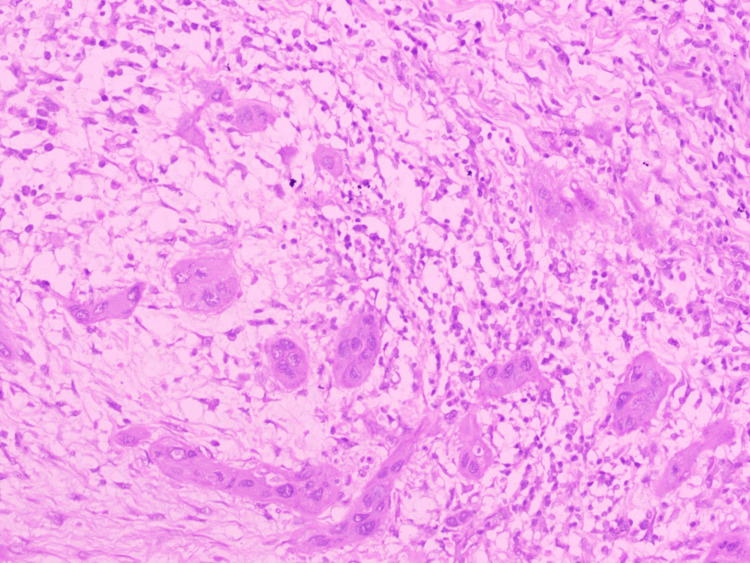
Hematoxylin and eosin stain (x400): tumor budding (high risk).

TB is defined as a single tumor cell or a cluster of <5 tumor cells in the stroma of the invasive front is defined as TB. The scoring that was used was a two-tier TB score: low risk (0-4 buds) and high risk (≥5 buds) (Figure 2) [7].

Tumor-infiltrating lymphocytes (TILs)

**Figure 3:**
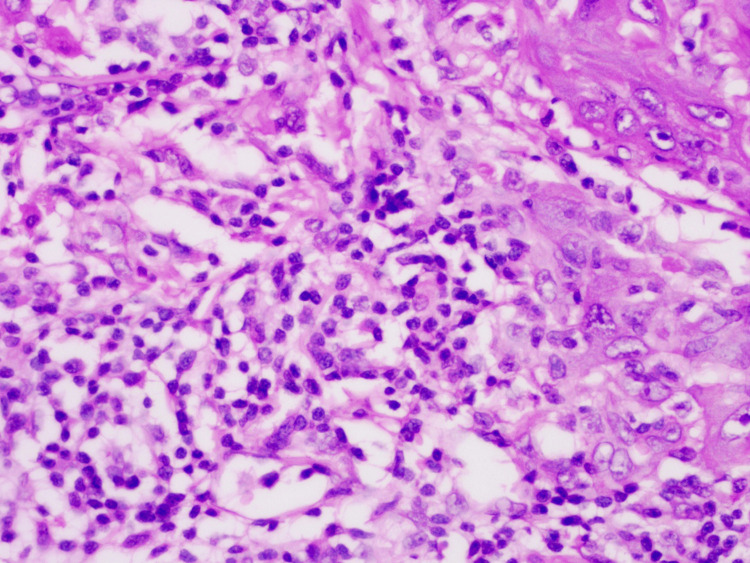
Hematoxylin and eosin stain (x400): tumor-infiltrating lymphocytes (high infiltration).

TILs represent the percentage of stromal TIL within the invasive tumor. Scoring was conducted based on stromal TIL: low infiltration (<20%) and high infiltration (≥20%) (Figure 3) [8].

Tumor-stroma ratio (TSR)

**Figure 4:**
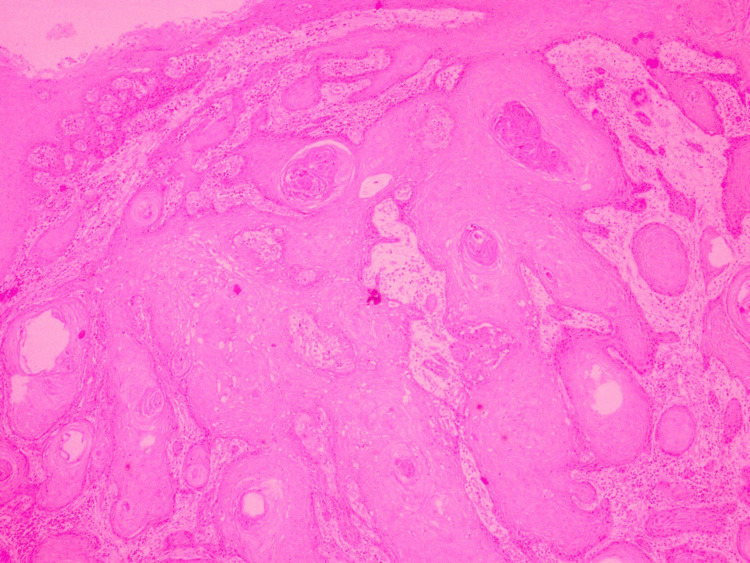
Hematoxylin and eosin stain (x200): tumor-stroma ratio (stroma poor).

TSR measures the proportion of tumor tissue relative to surrounding stromal tissue. It was scored as stroma-rich (≥50% stroma) and stroma poor (<50% stroma) (Figure 4) [9].

Worst pattern of invasion (WPOI)

Five patterns depend on the arrangement of tumor cells at the advancing front: Pattern 1 features a broad, pushing margin with a smooth outline. Pattern 2 displays an infiltrating, finger-like projection. Pattern 3 comprises tumor islands with >15 cells per island. Pattern 4 consists of tumor islands with <15 cells per island. Pattern 5 indicates the presence of a tumor island outside the main tumor at a distance of >1 mm [10].

Depth of invasion (DOI)

DOI refers to the distance from the basement membrane of the adjacent normal area to the deepest point of the invasion of the tumor scored as ≤5 mm, >5 to ≤10 mm, and >10 mm according to the T stage of TNM classification.

Perineural invasion (PNI)

**Figure 5:**
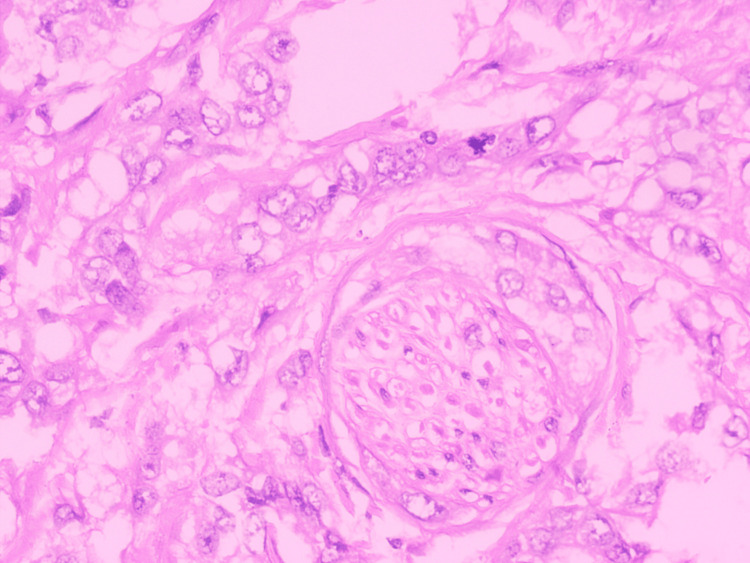
Hematoxylin and eosin (x400): perineural invasion.

PNI is a tropism of tumor cells for nerve bundles in the surrounding stroma and is reported as present or absent according to the College of American Pathologists (CAP) protocol (Figure 5).

Lymphovascular invasion (LVI)

**Figure 6:**
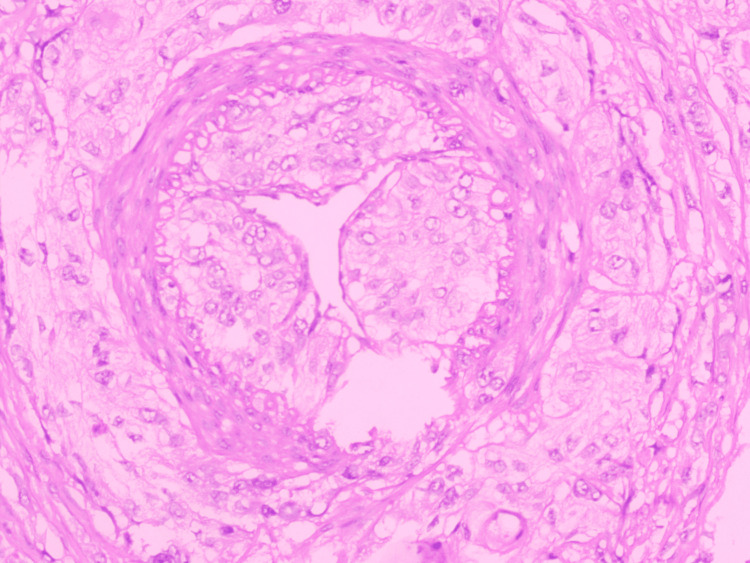
Hematoxylin and eosin stain (x400): lymphovascular invasion.

Lymphatic and vascular invasions are reported as present or absent according to CAP protocol (Figure 6).

Bone and skin invasion and regional lymph node metastasis were noted separately. All the recorded data were accumulated and statistically analyzed to determine the incidence occurrence in this pilot study group of 50 cases. Additionally, the study examined the correlation between each data point and lymph node metastasis, as well as the two-year survival rate.

## Results

We studied a total of 50 cases of OSCC, for which all the required clinical and histopathological details were available. Regional lymph node metastasis status was assessed in all 50 cases. Patient outcome was studied as overall two-year survival in 40 patients who were operated on at least two years ago. Ten patients out of 50 were lost to follow-up. Table 1 shows the overall results with clinical and histological details. 

**Table 1:** Overall results. SD, standard deviation

Parameter	Number/Mean	Percentage
Age (years)		
Mean ± SD	47.80 ± 12.48	
Range	29-76	
Gender		
Male	47	94.00%
Female	3	6.00%
Substance abuse		
Tobacco chewing	28	56.00%
Smoking	7	14.00%
Alcohol	4	8.00%
Procedure		
Bite composite	17	34.00%
Marginal mandibulectomy	2	4.00%
Partial glossectomy	6	12.00%
Segmental mandibulectomy	15	30.00%
Wide local excision of buccal mucosa	2	4.00%
Mixed	8	16.00%
Laterality of tumor		
Bilateral	1	2.00%
Left	30	60.00%
Right	19	38.00%
Site of tumor		
Alveolar process	5	10.00%
Buccal mucosa	19	38.00%
Gingivo-buccal sulcus	5	10.00%
Mixed	6	12.00%
Retro molar trigone	2	4.00%
Tongue	13	26.00%
Maximum dimension		
Mean ± SD	2.85 ± 1.08	
Range	0.80-6 cm	
2 cm	10	20.00%
>2-4 cm	35	70.00%
>4 cm	5	10.00%
Differentiation		
Moderate	14	28.00%
Poor	6	12.00%
Well defined	30	60.00%
Surgical margin		
Close	2	4.00%
Free	46	92.00%
Involved	2	4.00%
Tumor budding		
High risk	14	28.00%
Low risk	36	72.00%
Tumor-stroma ratio		
Stroma-poor	27	54.00%
Stroma-rich	23	46.00%
Tumor-infiltrating lymphocyte		
High infiltration	26	52.00%
Low infiltration	24	48.00%
Depth of invasion		
5 mm	6	12.00%
>5 to 10 mm	18	36.00%
>10 mm	26	52.00%
Worst pattern of invasion		
Pattern 2	1	2.00%
Pattern 3	8	16.00%
Pattern 4	25	50.00%
Pattern 5	16	32.00%
Perineural invasion		
Yes	24	48.00%
No	26	52.00%
Lymphovascular invasion		
Yes	19	38.00%
No	31	62.00%
Total number of lymph nodes dissected		
Mean ± SD	27.16 ± 10.61	
Range	9-62	
Extra-nodal extension		
No	10	20.00%
Yes	9	18.00%
Not applicable	31	62.00%
Bone invasion		
No	22	44.00%
Yes	13	26.00%
Not applicable	15	30.00%
Skin invasion		
No	7	14.00%
Yes	5	10.00%
Not applicable	38	76.00%
T stage		
Stage 1	4	8.00%
Stage 2	13	26.00%
Stage 3	16	32.00%
Stage 4	17	34.00%
N stage		
Stage 0 (No lymph node metastasis)	32	64.00%
Stage 1	8	16.00%
Stage 2	6	12.00%
Stage 3	4	8.00%
Two-year survival		
Dead	17	34.00%
Healthy	19	38.00%
Recurrence	4	8.00%
Lost to follow-up	10	20.00%

Based on the data presented in Table 1, the following observations were made regarding the key factors in demographics, risk factors, and clinical parameters. The mean age of patients with OSCC was 47.80 years, with a standard deviation of 12.48 years. The majority of patients were male (47, 94%), and the most common substance abused was tobacco chewing (56%). The most frequent surgical procedure performed was segmental mandibulectomy (30%), and the most common site of the tumor was the buccal mucosa (38%). The mean maximum tumor dimension was 2.85 cm, with the majority of tumors falling within the >2 to 4 cm category (70%).

Histological features were also evaluated and showed that most tumors were well-differentiated (60%) and had free surgical margins (92%). High-risk tumor budding was observed in 28% of cases, while 54% of tumors were categorized as stroma-poor. Over half of the tumors had a depth of invasion greater than 10 mm (52%), and the most common worst pattern of invasion was Pattern 4 (50%). PNI was present in 48% of cases, while LVI was observed in 38% of cases.

The staging was performed based on the AJCC 8th edition. The majority of tumors were classified as T3/T4 stage (66%), while most patients had no lymph node metastasis (64%). The two-year survival rate was 38%, with 34% of patients deceased, 8% experiencing recurrence, and 20% lost to follow-up. These findings provide valuable insights into the clinical and pathological characteristics of OSCC in the studied population, which may contribute to improved diagnosis, treatment, and prognosis for affected individuals.

Comparative results based on nodal metastasis status

The study compared various parameters between patients with lymph node metastasis (LN Mets +) and those without (LN Mets -) in cases of OSCC, as tabulated in Table 2.

**Table 2:** Comparative assessment of the regional lymph node metastasis. Bold values highlight *P*-values of 0.05 or lower, which are considered statistically significant.

Parameter	LN Mets +	%	LN Mets -	%	*P*-value
Number of patients	18		32		-
Age (years)					
Mean ± SD	46.67 ± 11.54		47.81 ± 13.14		0.5184
Range	31-70		29-76		-
Gender					
Female	0	0.00%	3	9.38%	0.1846
Male	18	100.00%	29	90.63%	
Substance abuse					
Tobacco chewing	14	77.78%	14	43.75%	0.0213
Smoking	0	0.00%	7	21.88%	0.0341
Alcohol	1	5.56%	3	9.38%	0.6362
Procedure					
Bite composite	5	27.78%	12	37.50%	0.4906
Marginal mandibulectomy	0	0.00%	2	6.25%	0.2839
Partial glossectomy	2	11.11%	4	12.50%	0.8857
Segmental mandibulectomy	5	27.78%	10	31.25%	0.7992
Wide local excision of buccal mucosa	5	27.78%	2	6.25%	0.0371
Mixed	6	33.33%	2	6.25%	0.0131
Laterality of tumor					
Bilateral	1	5.56%	0	0.00%	0.1822
Left	10	55.56%	20	62.50%	0.6341
Right	7	38.89%	12	37.50%	0.9233
Site of tumor					
Alveolar process	2	11.11%	3	9.37%	0.2654
Buccal mucosa	5	27.78%	14	43.75%	0.2476
Gingivo-buccal sulcus	0	0.00%	5	15.63%	0.0801
Mixed	2	11.11%	4	12.50%	0.6517
Retro molar trigone	1	5.56%	1	3.12%	0.6771
Tongue	8	44.44%	5	15.63%	0.0273
Maximum dimension					
Mean ± SD	3.32 ± 1.21		2.59 ± 0.92		0.1844
Range	1.50-6 cm		0.80-4.50 cm		-
2 cm	2	11.11%	8	25.00%	0.2433
>2 to 4 cm	12	66.67%	23	71.88%	0.7024
>4 cm	4	22.22%	1	3.13%	0.0325
Differentiation					
Well	7	38.89%	23	71.88%	0.0237
Moderate	8	44.44%	6	18.75%	0.0545
Poor	3	16.67%	3	9.38%	0.4511
Surgical margin					
Close	1	5.56%	1	3.13%	0.6771
Free	16	88.89%	30	93.75%	0.5472
Involved	1	5.56%	1	3.13%	0.6771
Tumor budding					
High risk	4	22.22%	10	31.25%	0.4992
Low risk	14	77.78%	22	68.75%	-
Tumor-stroma ratio					
Stroma-poor	7	38.89%	20	62.50%	0.1115
Stroma-rich	11	61.11%	12	37.50%	-
Tumor-infiltrating lymphocyte					
High infiltration	6	33.33%	20	62.50%	0.0498
Low infiltration	12	66.67%	12	37.50%	-
Depth of invasion					
5 mm	1	5.56%	5	15.63%	0.2979
>5 to 10 mm	6	33.33%	12	37.50%	0.7704
>10 mm	11	61.11%	15	46.88%	0.3386
Worst pattern of invasion					
Pattern 2	0	0.00%	1	3.13%	0.4529
Pattern 3	2	11.11%	6	18.75%	0.4838
Pattern 4	11	61.11%	14	43.75%	0.2434
Pattern 5	5	27.78%	11	34.38%	0.6345
Perineural invasion					
Yes	13	72.22%	11	34.38%	0.0109
No	5	27.78%	21	65.63%	-
Lymphovascular invasion					
Yes	14	77.78%	5	15.63%	<0.0001
No	4	22.22%	27	84.38%	-
Total no. of lymph nodes dissected					
Mean ± SD	29.06 ± 12.65		26.09 ± 9.32		0.5178
Range	9-62		14-48		
Extra-nodal extension					
No	9	50.00%	1	3.13%	0.0001
Yes	9	50.00%	0	0.00%	<0.0001
Not applicable	0	0.00%	31	96.88%	<0.0001
Bone invasion					
No	5	27.78%	17	53.13%	0.0862
Yes	5	27.78%	8	25.00%	0.8314
Not applicable	8	44.44%	7	21.88%	0.0981
Skin invasion					
No	1	5.56%	6	18.75%	0.2015
Yes	2	11.11%	3	9.38%	0.8464
Not applicable	15	83.33%	23	71.88%	0.3677
T stage					
Stage 1	0	0.00%	4	12.50%	0.1216
Stage 2	3	16.67%	10	31.25%	0.2641
Stage 3	7	38.89%	9	28.13%	0.4383
Stage 4	8	44.44%	9	28.13%	0.2473

The study comprised 50 patients with OSCC. Eighteen patients (36%) were seen to have nodal metastasis in the study. Among the 18 patients with nodal metastasis in the study, the majority were N1 patients (8, 16%), followed by N2 patients (6, 12%). N3 was seen in 4 patients (8%) in the study.

A comparative assessment of regional lymph node metastasis with various clinical and histological parameters was done, and the results were noted. It was observed that the youngest and oldest patients in the study were 29 and 76 years old, respectively. The average age of patients was 47.80 ± 12.48 years. It was interesting to note that the average age of patients with nodal metastasis was slightly lower compared to those without nodal metastasis (46.67 versus 47.81 years). The *P*-value was 0.5184. Seventy-eight percent of patients in the study had a history of substance abuse (tobacco/alcohol/smoking). The trends showed significant *P*-value among tobacco abusers and smokers. Most of the patients had a maximum tumor dimension of >2 to 4 cm (70%) followed by those with a maximum dimension of <2 cm (20%). Ten percent of patients had a maximum tumor dimension of >4 cm. The average maximum dimension of the lesion was 2.85 ± 1.08 cm. The lesions with nodal metastasis had a higher maximum dimension compared to the patients without nodal metastasis (3.32 cm versus 2.59 cm). The difference was significant statistically in those with tumor dimensions >4 cm. The most common tumor site was buccal mucosa (38%) followed by the tongue (26%). An important observation was that the proportion of the tongue as the tumor site was significantly higher in the patients with nodal metastasis compared to those without a nodal metastasis lesion (44.44% versus 15.63%). The *P*-value was 0.0273.

Among the histological parameters, the proportion of well-differentiated tumors was significantly higher in the patients with no nodal metastasis (71.88% versus 38.89% in nodal metastasis, *P *= 0.0237). It was interesting to see that the patients with nodal metastasis had a significantly higher proportion of patients with low tumor infiltration of lymphocytes (66.67%) than high infiltration (14.81%) with a P value of 0.0498 suggestive of statistical significance. The PNI and LVI were significantly higher for the patients with nodal metastasis. The extra-nodal extension was seen in 50% of the patients with nodal metastasis.

These findings highlight several clinical, epidemiological, risk factors, histopathological, and invasive factors associated with lymph node metastasis in patients with OSCC.

As a second part of section 1, a Spearman correlation coefficient-based univariate analysis for the predictors of nodal metastasis was done and tabulated in Table 3. It was seen that tobacco chewing had a positive whereas smoking had a negative correlation with nodal metastasis. The tumor with a higher maximum size dimension had higher nodal metastasis. Differentiation was another significant parameter. It was seen that poor differentiation was positively correlated with nodal metastasis with statistically significant results (*P *= 0.0315). The lower the infiltration of lymphocytes in the tumor, the higher the lymph node metastasis. Both PNI and LVI showed a significant positive correlation with the occurrence of lymph node metastasis.

**Table 3:** Univariate predictors of nodal metastasis.

LN Mets	Rho	95% CI	*P*-value	N
Age	-0.036	-0.32 to 0.25	0.8033	50
Gender	0.19	-0.10 to 0.45	0.1875	50
Tobacco chewing	0.33	0.047 to 0.56	0.0196	50
Smoking	-0.3	-0.54 to -0.018	0.0327	50
Alcohol	-0.068	-0.35 to 0.22	0.641	50
Maximum dimension (cm) (A = 0, B = 1, C = 2)	0.29	0.00083 to 0.53	0.0434	50
Differentiation (WD = 0, MD = 1, PD = 2)	0.3	0.020 to 0.54	0.0315	50
Surgical margin (Free = 0, Close/Involved = 1)	0.086	-0.21 to 0.36	0.5526	50
Tumor budding (LR = 0, HR = 1)	-0.097	-0.37 to 0.19	0.5049	50
Tumor-stroma ratio (SR = 0, SP = 1)	-0.23	-0.48 to 0.063	0.1123	50
Tumor-infiltrating lymphocytes (LI = 0, HI = 1)	-0.28	-0.52 to 0.0064	0.0487	50
Depth of invasion (A = 0, B = 1, C = 2)	0.16	-0.13 to 0.43	0.2662	50
Worst pattern of invasion	0.02	-0.27 to 0.30	0.8877	50
Perineural invasion (No = 0, Yes = 1)	0.36	0.086 to 0.59	0.0094	50
Lymphovascular invasion (No = 0, Yes = 1)	0.61	0.40 to 0.77	<0.0001	50
Total number of lymph nodes dissected	0.12	-0.17 to 0.39	0.401	50
Involved lymph nodes	0.91	0.84 to 0.95	<0.0001	50
Extra-nodal extension (No = 0, Yes = 1)	0.22	-0.27 to 0.62	0.3574	19
Bone invasion (No = 0, Yes = 1)	0.17	-0.18 to 0.48	0.3339	35
Skin invasion (No = 0, Yes = 1)	0.29	-0.35 to 0.75	0.5227	12
T stage	0.27	-0.020 to 0.51	0.06	50

Comparative results based on overall two-year survival

At the end of the follow-up period (overall two-year survival) for 50 patients, 17 experienced mortality (34%), recurrence was seen in 4 patients (8%), and 19 patients (38%) were healthy and alive at the end of the follow-up period, suggesting an overall two-year survival rate of 38%. However, 10 patients were lost to follow-up (Table 4).

**Table 4:** Overall two-year analysis.

Overall outcome	Number	Percentage
Dead	17	34%
Healthy	19	38%
Recurrence	4	8%
Lost to follow-up	10	20%
Grand total	50	100%

According to the comparison of two-year survival, each prognostic entity under the study is tabulated in Table 5. The following findings were noted: Notably, none of the patients with tumor dimension less than or equal to 2 cm experienced mortality in a two-year follow-up period (*P*-value = 0.0134), whereas all four patients with more than 4 cm tumor size succumbed to disease (*P*-value = 0.0155). A significant number of survivors had a well-differentiated tumor morphology (*P*-value = 0.0023). In contrast, almost half of the diseased individuals had a moderately differentiated tumor (*P*-value = 0.0187). The majority of healthy patients had a high tumor infiltration of lymphocytes (78.26%), whereas infiltration in a significant number of dead individuals was low (82.35%), with a combined *P*-value of 0.0002. A significant number of survivors had no lymph node metastasis (82.62%, *P*-value = 0.0073). A substantial proportion of patients who had nodal metastasis with tumors extending beyond the lymph nodes were reported to be dead (*P*-value = 0.0163) (Table 5).

**Table 5:** Comparative assessment for the two-year survival status. Note: Patients lost to follow-up were excluded. Bold values highlight *P*-values of 0.05 or lower, which are considered statistically significant. SD, standard deviation

Parameter	No mortality	%	Mortality	%	*P*-value
Number of patients	23		17		-
Age (years)					
Mean ± SD	50.83 ± 13.10		43.41 ± 11.82		0.0771
Range	31-76		29-71		-
Gender					
Male	3	13.04%	0	0.00%	0.1264
Female	20	86.96%	17	100.00%	-
Substance abuse					
Tobacco chewing	13	56.52%	8	47.06%	0.5587
Smoking	3	13.04%	4	23.53%	0.3940
Alcohol	0	0.00%	3	17.65%	0.0386
Procedure					
Bite composite	6	26.09%	6	35.29%	0.5354
Marginal mandibulectomy	0	0.00%	2	11.76%	0.0957
Partial glossectomy	1	4.35%	4	23.53%	0.0734
Segmental mandibulectomy	5	21.74%	7	41.18%	0.1903
Wide local excision of buccal mucosa	6	26.09%	1	5.88%	0.1006
Mixed	5	21.74%	3	17.65%	0.7523
Laterality of tumor					
Bilateral	0	0.00%	1	5.88%	0.2449
Left	13	56.52%	9	52.94%	0.8242
Right	10	43.48%	7	41.18%	0.8858
Site of tumor					
Alveolar process	4	17.39%	1	5.88%	0.2826
Buccal mucosa	7	30.44%	7	41.18%	0.4870
Gingivo-buccal sulcus	4	17.39%	0	0.00%	0.0735
Mixed	2	8.70%	2	11.76%	0.7528
Retro molar trigone	0	0.00%	1	5.88%	0.2449
Tongue	6	26.09%	6	35.29%	-
Maximum dimension					
Mean ± SD	2.44 ± 0.92		3.29 ± 0.97		0.0055
Range	0.80-4 cm		1.80-5 cm		
2 cm	7	30.43%	0	0.00%	0.0134
>2-4 cm	16	69.57%	13	76.47%	0.6333
>4 cm	0	0.00%	4	23.53%	0.0155
Differentiation					
Well	18	78.26%	5	29.41%	0.0023
Moderate	3	13.04%	8	47.06%	0.0187
Poor	2	8.70%	4	23.53%	0.1998
Surgical margin					
Close	1	4.35%	1	5.88%	0.8284
Free	21	91.30%	15	88.24%	0.7528
Involved	1	4.35%	1	5.88%	0.8284
Tumor budding					
High risk	6	26.09%	6	35.29%	0.5354
Low risk	17	73.91%	11	64.71%	
Tumor-stroma ratio					
Stroma poor	13	56.52%	8	47.06%	0.5587
Stroma rich	10	43.48%	9	52.94%	
Tumor-infiltrating lymphocyte (TIL)					
High infiltration	18	78.26%	3	17.65%	0.0002
Low infiltration	5	21.74%	14	82.35%	
Depth of invasion (DOI)					
5 mm	4	17.39%	1	5.88%	0.2826
>5 to 10 mm	9	39.13%	4	23.53%	0.3038
>10 mm	10	43.48%	12	70.59%	0.0925
Worst pattern of invasion					
Pattern 2	0	0.00%	1	5.88%	0.2449
Pattern 3	4	17.39%	2	11.76%	0.6264
Pattern 4	12	52.17%	8	47.06%	0.7524
Pattern 5	7	30.43%	6	35.29%	0.7487
Perineural invasion					
Yes	10	43.48%	11	64.71%	0.1894
No	13	56.52%	6	35.29%	
Lymphovascular invasion					
Yes	6	26.09%	10	58.82%	0.0392
No	17	73.91%	7	41.18%	
Total no. of lymph nodes dissected					
Mean ± SD	27.83 ± 10.53		28.82 ± 11.78		0.7714
Range	15-49		14-62		-
Extra-nodal extension					
No	3	13.04%	3	17.65%	0.6902
Yes	2	8.70%	7	41.18%	0.0163
Not applicable	18	78.26%	7	41.18%	0.0181
Bone invasion					
No	9	39.13%	7	41.18%	0.8972
Yes	7	30.43%	4	23.53%	0.6333
Not applicable	7	30.43%	6	35.29%	0.7487
Skin invasion					
No	3	13.04%	3	17.65%	0.6902
Yes	1	4.35%	2	11.76%	0.3851
Not applicable	19	82.61%	12	70.59%	0.3742
T (tumor) stage					
Stage 1	2	8.70%	1	5.88%	0.7410
Stage 2	8	34.78%	2	11.76%	0.1007
Stage 3	5	21.74%	7	41.18%	0.1903
Stage 4	8	34.78%	7	41.18%	0.6832
N (nodal) stage					
Stage 0 (LN Mets -)	19	82.61%	7	41.18%	0.0073
LN Mets +	4	17.39%	10	58.82%	
Stage 1	2	8.70%	3	17.65%	-
Stage 2	0	0.00%	5	29.41%	-
Stage 3	2	8.70%	2	11.76%	-

Furthermore, a Spearman correlation coefficient-based univariate analysis was conducted to assess the predictors of survival. It was seen that the maximum dimension of the tumor was positively related to mortality: the higher the tumor size, the higher the mortality. Poorly differentiated morphology of tumors showed significantly poor survival in patients. Higher TILs are a favorable entity with better survival. LVI in tumors had a poor outcome in patients. Notably, the greater the involvement of lymph nodes by the tumor, the poorer the outcome (Table 6).

**Table 6:** Univariate predictors of overall two-year survival. WD, well differentiated; MD, moderately differentiated; PD, poorly differentiated; LR, low risk; HR, high risk; SR, stroma-rich; SP, stroma poor; LI, low infiltration; HI, high infiltration; T stage, tumor stage according to the American Joint Committee on Cancer (AJCC) 8th edition.

Mortality	Rho	95% CI	*P*-value	N
Age	-0.3	-0.57 to 0.019	0.0577	40
Sex	0.24	-0.082 to 0.52	0.1279	40
Tobacco chewing	-0.094	-0.40 to 0.23	0.5653	40
Smoking	0.14	-0.19 to 0.44	0.4012	40
Alcohol	0.33	0.012 to 0.59	0.0368	40
Maximum dimension (cm) (A = 0, B = 1, C = 2)	0.51	0.23 to 0.71	0.0007	40
Differentiation (WD = 0, MD = 1, PD = 2)	0.46	0.17 to 0.68	0.0027	40
Surgical margin (Free = 0, Close/Involved = 1)	0.051	-0.27 to 0.36	0.7566	40
Tumor budding (LR = 0, HR = 1)	0.099	-0.23 to 0.41	0.542	40
Tumor-stroma ratio (SR = 0, SP = 1)	-0.094	-0.40 to 0.23	0.5653	40
Tumor-infiltrating lymphocytes (LI = 0, HI = 1)	-0.6	-0.77 to -0.35	<0.0001	40
Depth of invasion (A = 0, B = 1, C = 2)	0.28	-0.047 to 0.55	0.0834	40
Worst pattern of invasion	0.026	-0.30 to 0.34	0.8719	40
Perineural invasion (No = 0, Yes = 1)	0.21	-0.12 to 0.50	0.1931	40
Lymphovascular invasion (No = 0, Yes = 1)	0.33	0.011 to 0.59	0.0374	40
Total number of lymph nodes dissected	0.031	-0.29 to 0.35	0.8508	40
Involved lymph nodes	0.37	0.052 to 0.61	0.0201	40
Extra-nodal extension (No = 0, Yes = 1)	0.29	-0.28 to 0.71	0.5804	15
Bone invasion (No = 0, Yes = 1)	-0.074	-0.45 to 0.33	0.7143	27
Skin invasion (No = 0, Yes = 1)	0.16	-	>0.9999	9
T stage	0.18	-0.15 to 0.47	0.2801	40

## Discussion

OSCC is a prevalent form of head and neck cancer that arises in the oral mucosa. GCO data from 2020 revealed 377,713 cases of OSCC worldwide, predominantly concentrated in Asia [11]. The global incidence of OSCC varies, with India and Southeast Asia having the highest rates. Each year, there are over 50,000 new cases reported globally, with a majority of patients being diagnosed at an advanced disease stage [12]. The tongue, specifically its movable section, is the most frequent and lethal location for OSCC [13]. Despite significant research and advancements in the field, OSCC continues to present significant challenges with its notably high morbidity and mortality rates.

The American Joint Committee on Cancer (AJCC) and the Union for International Cancer Control (UICC) TNM staging systems are extensively employed worldwide to ensure consistent cancer reporting, prognosis assessment, and treatment planning. Moreover, diverse histopathological risk assessment predictors, including tumor thickness (TT), tumor shape, growth pattern, and invasive malignancy grading systems, are utilized to predict and determine prognosis.

Nonetheless, given the constraints on the depth and size of biopsy samples, it is essential to have a dependable technique for precisely evaluating histopathological parameters. Thus, integrating the evaluation of various histomorphological parameters with clinical staging may present a fresh, systematic method for assessing the progression and prognosis of the disease. This combined strategy has the potential to improve the precision of prognostic predictions and assist clinicians in making treatment decisions for individuals with OSCC.

In this study, we examined 50 cases of OSCC in Eastern Uttar Pradesh, comprehensively analyzing demographic, clinical, and histopathological parameters to elucidate their correlations with regional lymph node metastasis and overall two-year survival outcomes.

Our findings revealed several noteworthy observations regarding the demographic and risk factors associated with OSCC. The mean age of patients with OSCC in our cohort was 48 years, consistent with global trends. Our study also highlights a male predominance. In our study, the most common tumor site was buccal mucosa (38%) followed by the tongue (26%). Clinical features such as the location and size of the tumor are crucial factors in determining the progression and prognosis of the disease. Our observations revealed that the buccal mucosa was the most frequent site of tumor development, followed by the tongue, contrary to the literature, which states that the tongue is the most common site [14].

However, these findings in the study, mirror findings from a few analogous Indian studies. Khan et al. also showed that OSCC was more common in males in the fifth to seventh decade of their life, with the most common site being buccal mucosa and tongue [15]. Sharma et al. in a retrospective study on trends in epidemiology of OSCC in Western Uttar Pradesh studied 80 cases from the period of 2004 to 2009. They observed a male-to-female ratio of 2.2:1 among patients with OSCC, along with a significant association with tobacco consumption. Additionally, the most common site of the tumor was noted to be the buccal mucosa [16].

There is a consistent finding in various research studies that OSCC is more prevalent in males than in females. This indicates that gender may have a notable impact on the occurrence of OSCC, potentially due to higher exposure to substance abuse in India. OSCC typically affects individuals in the middle to older age groups, and our research group shows a lower average age group compared to the other studies. This suggests that age is a contributing factor to the development of OSCC, with the risk increasing as individuals age. The consumption of tobacco is closely linked to OSCC. This underscores a strong connection between tobacco usage and the onset of oral cancer, underscoring the importance of implementing tobacco control measures to decrease the prevalence of OSCC. These demographic characteristics are consistent with established risk factors for OSCC, underscoring the ongoing significance of preventive strategies aimed at tobacco cessation and alcohol consumption.

In general, these results emphasize the significance of focused preventive actions, particularly among high-risk populations like middle-aged to elderly males who have a notable history of tobacco consumption. These measures should aim to lessen the impact of OSCC and enhance public health outcomes.

Furthermore, OSCC metastasis is characterized by its ability to spread easily through draining lymphatics. It is not very common for OSCC to metastasize to distant sites. However, there is still debate regarding whether clinically zero nodal status in patients with OSCC should be an indication for elective neck dissection in all patients. The traditional methods of T (tumor) staging and pathological grading are showing limitations in predicting metastasis. In this investigation, we also aim to examine different histological factors that could assist in indicating potential nodal metastasis and predicting prognosis and recurrence [17-20].

In our study, lymph node dissection was performed in all 50 cases and the study concludes that the nodal metastasis rate is 36%. Akhter et al. observed that the rate of lymph node metastasis in their study was 30%. They also showed that other comparable studies have a nodal metastasis rate of 35% to 60% [21]. Thus, the regional lymph node metastasis rate in our study was comparable to these observed numbers, indicating the reliability and consistency of our findings within the broader literature. The average age for patients with nodal metastasis was slightly lower compared to those without nodal metastasis (46.67 versus 47.81 years). This observation emphasizes the importance of considering age as part of risk stratification and treatment planning for patients with OSCC.

An important observation in our study was that the proportion of the tongue as the tumor site was significantly higher in patients with nodal metastasis compared to those without a nodal metastasis lesion (44.44% versus 15.63%). The tongue is situated anatomically on the base of the oral cavity, an area known for its abundant lymphatic vessels and neurovascular bundles. Consequently, patients with tongue cancers are more prone to nodal metastasis. Conversely, cancer in the buccal mucosa exhibits a lower incidence of lymph node metastasis [22].

The lesions with nodal metastasis had a higher maximum dimension compared to the patients without nodal metastasis (3.32 cm versus 2.59 cm). The difference was significant statistically in those with tumor dimensions >4 cm. The greatest dimension of the tumor surface is used to indicate tumor size in the TNM classification. Disagreements arise concerning this factor, with certain authors proposing a correlation between larger tumor size and cervical nodal metastasis, whereas others suggest that tumor size is not a reliable predictor of nodal metastasis [23-26].

The proportion of well-differentiated tumors was significantly higher in the patients with no nodal metastasis (71.88% versus 38.89% in nodal metastasis, *P *= 0.0237). Khan et al. showed that a significant relation was evident between histological grades and cervical lymph node metastasis, suggesting that higher grades of OSCC have a higher risk of metastatic lymph nodes, similar to our study. The study states concordant facts as proven multiple times that differentiation or the tumor grade plays a role in nodal metastasis [27].

It was interesting to see that the patients with nodal metastasis had a significantly higher proportion of patients with low tumor infiltration of lymphocytes (66.67%) than high infiltration (14.81%), with a *P*-value of 0.0498 suggestive of statistical significance. Yadav et al. in their study showed that the risk of metastases to cervical lymph nodes in patients with lymphocyte predominance was less (28.6%), and these results were statistically significant (*P* < 0.05), as seen in our study as well [28].

In our research, it is evident that patients with lower TIL levels exhibit a higher incidence of lymph node metastasis, serving as an indicator of both poor prognosis and increased likelihood of recurrence. TILs are mononuclear cells naturally infiltrating the solid tumor micro-environment, encompassing all immune cells present at the tumor site as well [29,30]. The history of TILs extends back over two centuries. In 1863, Virchow noted the presence of leukocytes in neoplastic tissues [31]. The prognostic value of lymphocytes close to tumor cells has been documented [32,33]. The quantification of TILs not only may enhance the available clinical cancer staging data but also serve as a reliable predictor of disease progression [34-36]. The intricate interplay between TIL and the tumor has undergone thorough examination in both mouse models of tumor growth and human tumor tissues [37,38]. The tumor microenvironment emerges from continual and evolving interactions between the developing tumor and the host immune system, tasked with immune surveillance. Initially, the tumor shields itself from immune cell elimination and gradually evolves mechanisms to suppress its activities. With tumor advancement, TIL within the tumor microenvironment progressively loses functionality, leading to an inability to impede tumor progression [39].

Furthermore, the PNI and LVI were significantly higher for the patients with nodal metastasis. The extra-nodal extension was seen in 50% of the patients with nodal metastasis.

The univariate analysis for nodal metastasis showed that tobacco consumption, higher maximum dimension, poor tumor differentiation, lower TIL, and presence of LVI and PNI were significant independent predictors of nodal metastasis in our study. Shegaonkar et al. also showed that among all the parameters evaluated in their study for the patients with OSCC, variables like habit (tobacco use) (*P* = 0.045), tumor size (*P* = 0.003), and PNI (*P* = 0.0001) emerged as independent prognosticators and significantly correlated to the lymph node status of the patients [40].

Overall, our findings contribute to a better understanding of the clinicopathological factors influencing nodal metastasis in OSCC and provide valuable insights for risk stratification, treatment planning, and prognostic assessment in clinical practice. Further research is warranted to validate these findings and explore potential therapeutic targets aimed at reducing nodal metastasis and improving outcomes in patients with OSCC.

Furthermore, the study revealed an overall survival rate of 46% in our study at the end of a two-year follow-up period. Notably, none of the patients with tumor dimensions less than or equal to 2 cm experienced mortality in a two-year follow-up period (*P*-value = 0.0134) whereas all four patients with more than 4 cm tumor size succumbed to disease (*P*-value = 0.0155). A significant number of survivors had a well-differentiated tumor morphology (*P*-value = 0.0023). In contrast, almost half of the diseased individuals had a moderately differentiated tumor (*P*-value = 0.0187). The majority of healthy patients had a high tumor infiltration of lymphocytes (78.26%), whereas infiltration in a significant number of dead individuals was low (82.35%), with a combined *P*-value of 0.0002. A significant number of survivors had no lymph node metastasis (82.62%, *P*-value = 0.0073). A substantial proportion of patients who had nodal metastasis with a tumor extending beyond the lymph nodes were reported to be dead (*P*-value = 0.0163).

An assessment of the predictors of mortality based on the univariate analysis revealed that a higher maximum tumor dimension, poorly differentiated tumors, lower TILs, the presence of LVI, the presence of PNI, and regional lymph node metastasis were significant univariate factors associated with a higher mortality rate.

Dolens et al. [41] in their meta-analysis on prognostic factors for poor outcome in OSCC showed that depth of invasion, extra-nodal extension, PNI, LVI, involvement of the surgical margins, bone invasion, TT, and pattern of invasion were correlated with an increased risk for poor survival. Chang et al. showed that the patients had five-year overall survival and progression-free survival rates of 60.0% and 47.9%, respectively [42]. This was slightly higher compared to our study where overall survival is 46%. Suresh et al. [4] in their study on prognostic factors in patients with OSCC showed that age <65 years, female patients, alveolus lesion and tongue lesion, early T stage, and N0 had a significant positive impact on disease-free and overall survival of oral cancer patients. Our study was also able to show that the N0 stage was a significant predictor of overall survival.

Our study provides valuable insights into the prognostic factors influencing overall survival in patients with OSCC, shedding light on significant clinicopathological associations and their implications for clinical management.

At the end of the two-year follow-up period, our study reported an overall survival rate of 46%, reflecting the challenges in managing OSCC and the need for improved therapeutic approaches. Notably, tumor size emerged as a critical determinant of survival, with patients having smaller tumors (≤2 cm) experiencing significantly better survival outcomes compared to those with larger tumors. Conversely, all patients with tumors exceeding 4 cm succumbed to the disease, underscoring the prognostic significance of tumor size in OSCC.

Our findings also highlight the importance of tumor differentiation and the immune microenvironment in influencing survival outcomes. Well-differentiated tumors were associated with better survival rates, while almost half of the deceased individuals had moderately differentiated tumors. Additionally, a higher proportion of healthy patients exhibited high tumor infiltration of lymphocytes, contrasting with a significant number of deceased individuals showing low infiltration. These findings suggest the potential prognostic value of tumor differentiation and immune response in OSCC.

The presence of nodal metastasis and disease extension beyond the lymph nodes emerged as significant predictors of mortality. Patients without nodal metastasis demonstrated notably better survival rates, emphasizing the critical role of nodal status in prognostic assessment and treatment planning in OSCC. Notably, a substantial proportion of patients with nodal metastasis and tumor extension beyond the lymph nodes succumbed to the disease, highlighting the importance of early detection and management of nodal involvement to improve survival outcomes.

Comparison with previous studies revealed variations in survival rates, with our study reporting a slightly lower overall survival rate compared to some prior investigations. This underscores the heterogeneity in patient populations, tumor characteristics, and treatment modalities across studies. However, our findings align with existing literature highlighting various prognostic factors associated with poor survival outcomes in OSCC, including tumor size, differentiation, lymph node metastasis, and invasion characteristics.

Our findings have important clinical implications for risk stratification, treatment selection, and prognostic assessment in patients with OSCC. Understanding the complex interplay of tumor characteristics and host factors is crucial for optimizing therapeutic approaches and improving survival rates in this patient population. Further research is warranted to validate these findings and explore novel therapeutic interventions aimed at enhancing survival outcomes in patients with OSCC.

Nevertheless, this brief pilot study highlights the necessity for a comprehensive multicentric study involving a larger study population. As demonstrated by this pilot study, several intriguing findings were uncovered, which could potentially assist in predicting the prognosis, recurrence, and lymph node status of patients. This study has the potential to contribute to the development of methods for assessing risk based on the primary diagnostic biopsy wedge in conjunction with radiological features, ultimately aiding in treatment decision-making.

## Conclusions

The study conducted on 50 patients of OSCC in Eastern Uttar Pradesh of North India demonstrates significant correlations among various histopathological and clinicopathological factors. Nevertheless, this preliminary study is constrained by a small number of participants and a restricted timeframe. The correlation between this study and prognostic assessment should be investigated through additional extensive multicentric studies conducted over a longer period. We intend to expand the scope of the study to include a larger group of participants and extend the duration of the research.

It is imperative to conduct further research to improve our understanding of the various prognostic factors and enhance clinical outcomes in the treatment of this widespread malignancy with such alarming mortality rates, as emphasized by our comprehensive analysis.
